# Supplementary material for: Video-Feedback Approach Improves Parental Compliance to Early Behavioral Interventions in Children with Autism Spectrum Disorders during the COVID-19 Pandemic: A Pilot Investigation
Source: Children (Basel). 2022 Nov 8;9(11):1710. doi: 10.3390/children9111710 (PMC9689261; doi:10.3390/children9111710)
Supplement: Supplementary file 1 [file children-09-01710-s001.zip › children-1967937-supplementary.pdf]

ASP CT / IRIB CNR Telehealth COVID19 survey

Interviewed parent________ Interview Date __ /__/___Child ID______Date of Birth__/__/__

Teleassistance Start Date ____ /___/___ Teleassistance End Date ___ /___/___

**SECTION I: COVID CARE BURDEN**		
1. Number of children in the family 1° 2° 3° 4° 5°+		
2. Are there other children with neurodevelopmental or other medical conditions to care for?	Yes (Please, specify)	No
3. Were there other family members to especially care for?	Yes	No
4. Did you keep working during the lockdown?	Yes	No
5. If you answered yes, please tell us if you worked:	Part - Time	Full - Time

**SECTION II: WORRIES (COVID RELATED EMOTIONAL DISTRESS)**		
1. Did you feel particularly worried or anxious during the lockdown?	Yes	No
2. Were you concerned about being infected?	Yes	No
3. Were you concerned about losing your job?	Yes	No
4. Were you concerned about the child's treatment interruption?	Yes	No
5. Were you concerned about changes in the child's daily routine?	Yes	No
6. Were you concerned about changes in your life-style?	Yes	No

**SECTION III: CHILD SLEEP DISTURBANCE**			
1. How difficult was it for your child to fall asleep?			
a. Very difficult	b. Quite difficult	c. A bit difficult	d. Not difficult at all
2. Did your child use to take a nap?			
Yes	No	Sometimes	
3. Did your child usually wake up during night hours?			
Yes	No	Sometimes	
4. If you answered yes or sometimes, please tell us what he/she used to do: …			
5. Did your child take melatonin or other kinds of drugs to sleep at night?		Yes	No

SECTION IV: CHILD FEEDING/EVACUATION DISTURBANCE			
1. Was your child selective in his/her food preferences?	Yes	No	Sometimes
2. If you answered yes or sometimes, please tell us what he/she used to eat: …			
3. Did your child suffer from constipation?	Yes	No	Sometimes

SECTION V: PARENTS' TELEHEALTH JUDGMENT		
1. Did you find some benefits from the teleassistance program?	Yes	No
2. How much did you find teleassistance a positive experience for your child's daily management?		
3. How much do you feel teleassistance supported you with child's caring?		
4. How much did teleassistance help you to cope with child's complex behaviors?		
5. How much do you feel you have learned about your child's development during the telerehabilitation?		
6. How much do you feel you have learned about your strategies to interact with your child during the telerehabilitation?		
7. How much do you feel teleassistance supported you emotionally?		
8. Would you like to continue with some telerehabilitation sessions now the lock-down is over?		
a. Yes, I would like to maintain the remote modality only<br>b. Yes, I would like to start a blended modality combining in person with remote sessions<br>c. No, I think in person treatment only is the best program		

9. Please justify your answer:	
a. In presence therapy is more effective	b. Teleassistance is time-saving
c. We have difficulties to use technology	d. We live far from the center
e. We like the chance of having a parent training from remote	f. Others, please specify: …
